# The effectiveness and cost-effectiveness of a group domestic abuse perpetrator programme: protocol for a randomised controlled trial

**DOI:** 10.1186/s13063-023-07612-6

**Published:** 2023-09-28

**Authors:** Karen Morgan, Mei-See Man, Rachael Bloomer, Madeleine Cochrane, Melissa Cole, Sandi Dheensa, Nathan Eisenstadt, Gene Feder, Daisy M. Gaunt, Rwth Leach, Rebecca Kandiyali, Sian Noble, Tim J. Peters, Beverly A. Shirkey, Helen Cramer

**Affiliations:** 1 Centre for Academic Primary Care, Population Health Sciences, Bristol Medical School, University of Bristol, Bristol, UK; 2 Norwich Clinical Trials Unit, Norwich Medical School, University of East Anglia, Norwich, UK; 3 Bristol Trials Centre, Bristol Medical School, University of Bristol, Bristol, UK; 4 Warwick Clinical Trials Unit, Warwick Medical School, University of Warwick, Coventry, UK; 5 Bristol Dental School, University of Bristol, Bristol, UK

**Keywords:** Domestic abuse perpetrator programmes, Batterer interventions, Domestic violence and abuse, Intimate partner abuse, Domestic violence and abuse perpetrators

## Abstract

**Background:**

In contrast to evidence for interventions supporting victim/survivors of domestic violence and abuse (DVA), the effectiveness of perpetrator programmes for reduction of abuse is uncertain. This study aims to estimate the effectiveness and cost-effectiveness of a perpetrator programme for men.

**Methods:**

Pragmatic two-group individually randomised controlled trial (RCT) with embedded process and economic evaluation. Five centres in southwest England and South Wales aim to recruit 316 (reduced from original target of 366) male domestic abuse perpetrators. These will be randomised 2:1 to a community-based domestic abuse perpetrator programme (DAPP) or usual care comparator with 12-month follow-up. Female partners/ex-partners will be invited to join the study.

The intervention for men comprises 23 weekly sessions of a group programme delivered in voluntary sector domestic abuse services. The intervention for female partners/ex-partners is one-to-one support from a safety worker. Men allocated to usual care receive no intervention; however, they are free to access other services. Their partners/ex-partners will be signposted to support services.

Data is collected at baseline, and 4, 8 and 12 months’ follow-up. The primary outcome is men’s self-reported abusive behaviour measured by the Abusive Behaviour Inventory (ABI-29) at 12 months. Secondary measures include physical and mental health status and resource use alongside the abuse measure ABI (ABI-R) for partners/ex-partners and criminal justice contact for men.

A mixed methods process evaluation and qualitative study will explore mechanisms of effectiveness, judge fidelity to the intervention model using interviews and group observations.

The economic evaluation, over a 1-year time horizon from three perspectives (health and social care, public sector and society), will employ a cost-consequences framework reporting costs alongside economic outcomes (Quality-Adjusted Life Years derived from EQ-5D-5L, SF-12 and CHU-9D, and ICECAP-A) as well as the primary and other secondary outcomes.

**Discussion:**

This trial will provide evidence of the (cost)effectiveness of a DAPP. The embedded process evaluation will further insights in the experiences and contexts of participants and their journey through a perpetrator programme, and the study will seek to address the omission in other studies of economic evaluations.

**Trial registration:**

ISRCTN15804282, April 1, 2019

**Supplementary Information:**

The online version contains supplementary material available at 10.1186/s13063-023-07612-6.

## Introduction

### Background and rationale {6a}

Domestic violence and abuse (DVA) is a violation of human rights that harms the physical and mental health of victim/survivors and their families [1, 2]. The mental health problems associated with DVA include depression, anxiety disorder, post-traumatic stress disorder and alcohol and substance misuse [3, 4]. Children affected by DVA are at risk of poor school achievement, externalising and internalising behaviour, and have poor adult mental health and increased risk of perpetrating or experiencing DVA as adults [4]. Both women and men suffer DVA (annual prevalence in England and Wales 6.9% women and 3.0% men aged 16 and over [5]), although its prevalence, frequency and severity are greater in women, as is sexual violence and coercive control [6]. The annual cost to the economy for DVA is high, estimated in England and Wales as over £66 billion in 2018. As a result of the physical and emotional harms experienced by victim/survivors, DVA costs include lost output due to time off work, costs to the health service, housing costs, criminal justice system costs and victim/survivor support services [7].

Given the harmful effects of DVA, evidence is needed for effective preventive interventions, including reduction of perpetration [8]. Hence, the National Institute for Health and Care Excellence (NICE) [9] recommendation for the commissioning and evaluation of ‘tailored interventions for people who perpetrate DVA’. While provision of such services remains patchy in the UK and is absent in many countries, there has been an increase in programmes focusing on both individual and group work with perpetrators in recent decades [10]. However, the effectiveness of perpetrator interventions is uncertain [11–13]. Vigurs et al. [14] note that evidence regarding the effectiveness of DVA perpetrator interventions is ‘equivocal’ although Gondolf [15] reported evidence of reduced physical violence in those attending four well-established perpetrator interventions across the USA. The uncertainty about programme effectiveness arises from variations in research methodologies, programme design, lack of clarity about attrition from groups, outcomes and the difficulty in identifying specific elements of programme content that might contribute to behaviour change due to insufficient focus on these elements by programme evaluations [16–18]. Following their review of European evaluations, Lilley-Walker et al. [17] recommend that a more robust study of perpetrator interventions would require a range of elements including a control group design, a larger sample than that found in most evaluations, a comprehensive range of outcome measures, inclusion of data from partner victim/survivors and qualitative, as well as quantitative, methodologies. Where there is reporting of effectiveness in evaluations, the effect sizes are small and of questionable value. Consequently, although Project Mirabal did reveal encouraging ‘steps towards change’ for men attending domestic abuse perpetrator programmes (DAPPs) [19], many UK local authority commissioners have been slow to fund DAPPs because the evidence base is unconvincing.

To address the uncertainty about the effectiveness and cost-effectiveness of DAPPs and to address the lack of methodological clarity and other problems found in existing studies [17, 18], the REPROVIDE study has been designed as a pragmatic, individually randomised controlled trial with an embedded process evaluation. Given the anticipated challenges of recruitment and retention of perpetrators and their partners or ex-partners—hereafter referred to as (ex)partners—to a perpetrator programme randomised trial, we conducted a pilot study before progressing to the full trial. This ascertained that it is possible to recruit, randomise and retain both male perpetrators and their female partners/ex-partners, to collect the outcome data, and that the trial design is broadly acceptable [20].

Since the 1980s, group approaches ranging from voluntary support groups to structured interventions in prisons has been the most common form of DAPPs [19, 21]. Group interventions are more common because of the opportunities for peer learning, with participants more ready to change able to help others not yet at that stage [22–25]. Most programmes combine structured, feminist psycho-educational approaches (also referred to as the Duluth model [26]) combined with cognitive behavioural therapy and empathy building [19, 21, 27]. Positive changes reported for perpetrator programmes include better communication, parenting and interpersonal relationships, more responsibility for behaviour and control of behaviour, more empathy and self-awareness, less aggression and improved skills development [28].

Given the empirical and theoretical controversy over perpetrator intervention models [29] and in line with the Medical Research Council (MRC) framework for evaluation of complex interventions [30, 31], we developed our perpetrator intervention based on existing evidence and in consultation with a range of practitioner, researcher and policy stakeholders, including key collaborators Respect, a UK membership body and leaders in perpetrator work (https://www.respect.uk.net/). We also involved patient and public involvement (PPI) groups—specifically, women who had experienced domestic abuse and men who had completed a programme for domestic abuse.

### Objectives {7}

The primary aim of REPROVIDE is to estimate the effectiveness and cost-effectiveness of a group DAPP on men’s abusive behaviour.

### Trial design {8}

This is a multi-centre, two-group, pragmatic, individually randomised controlled trial with embedded process evaluation and economic evaluation. The bespoke online randomisation system is provided by the Bristol Trials Centre. Participants are randomly allocated in a 2:1 ratio to intervention or control respectively. Randomisation is stratified by centre (Bristol/North Somerset/South Gloucestershire (BNSSG), Somerset, Wiltshire, Blaenau Gwent and Neath Port Talbot) and minimised by relationship status (whether or not the participant is still living all or most of the time with the abused partner).

## Methods: participants, interventions and outcomes

### Study setting {9}

The community-based trial is taking place in three centres in the southwest of England (BNSSG, Somerset and Wiltshire) and two centres in South Wales (Blaenau Gwent, and Neath Port Talbot).

### Eligibility criteria {10}

Participants of the trial are male perpetrators of abuse aged 21 and over, who can speak English well enough to participate in a group intervention, and who recognise a need to change some abusive behaviours, and their female (ex)partners aged 18 and over (see Tables 1 and 2). Although male recruitment into the study is not conditional upon female (ex)partner’s agreement to participate, the research team must be provided with her contact details. This is to ensure that she is signposted to appropriate support or, if the male recruit has been randomised to the intervention arm, for the intervention service to be able to provide integrated support and to manage risk in accordance with Respect standards [10]. The requirement for contact details means that the research team can also ensure she is offered the opportunity to participate in the study, allowing measurement of (ex)partner outcomes and articulating their experience through interviews.

**Table 1:** Inclusion and exclusion criteria: male participants

**Inclusion criteria: male participants**
• 21 years of age
• Men who have used abusive behaviour in relationships with female (ex)partner(s)
• Ability to complete questionnaires with/without assistance of researcher
• Ability to participate in an English-speaking group setting
• Contact with an abused (ex)partner within the last 12 months at the time of recruitment or, anticipate having contact with an abused (ex)partner within the next 12 months.
**Exclusion criteria: male participants**
• Court mandated referral to a perpetrator programme
• Men who are deemed too high risk as assessed by a DAPP coordinator or by the research team
• Men who are deemed by the DAPP coordinator as unwilling to engage with the intervention
• Men with known previous violence or aggression towards professionals
• Participants who cannot understand English sufficiently well to give informed consent and to complete the questionnaires (with/without assistance) or to participate in a group setting
• Attendance on a group perpetrator programme consisting of more than 10 sessions in the previous 12 months
• Participants unable to consent to and engage with a group programme (includes, but is not limited to, persons with a serious mental health difficulty, serious learning disability or unstable substance or alcohol use)
• Men currently in Child Arrangement Order (CAO) proceedings with an open Children and Family Court Advisory and Support Service (CAFCASS) case, who have been in such proceedings in the last 12 months, or who state they intend to open such proceedings in the next 12 months
• Men who have ongoing criminal justice investigations for a DVA incident towards an (ex)partner (i.e., waiting to hear if will be going to court or waiting for a court date)
• Men who are unwilling or unable to provide (ex)partner contact details
• Men who fall outside the catchment areas of the regional police forces that correspond with the intervention locations: Avon and Somerset Police (Bristol, South Gloucestershire, North Somerset and Somerset), Wiltshire Police (Wiltshire), Gwent Police (Blaenau Gwent) and South Wales Police (Neath Port Talbot).

**Table 2:** Inclusion and exclusion criteria: female participants

**Inclusion criteria: female participants**
• 18 years of age
• Female (ex)partner of men using abusive behaviour in the relationship
• Ability to complete questionnaires with/without assistance of researcher.
**Exclusion criteria: female participants**
• Participants who cannot understand English sufficiently well to give informed consent and to complete the questionnaires (with/without assistance)
• Women who are deemed (by the women’s safety worker, DAPP coordinator or research team) to be at greater risk if they take part in the study.

### Who will take informed consent? {26a}

Informed consent will be obtained by a member of the research team and this will be checked and monitored by the programme manager.

### Additional consent provisions for collection and use of participant data and biological specimens {26b}

Not applicable. This study does not involve the collection and use of biological specimens.

## Interventions

### Explanation for the choice of comparators {6b}

Control group participants will receive usual care. Given the uncertainty about the effectiveness of group programmes, usual care in the form of any domestic abuse services available to perpetrators is deemed the most appropriate comparator. No group programme will be provided via the study.

### Intervention description {11a}

#### Development of the intervention

**Fig. 1:**
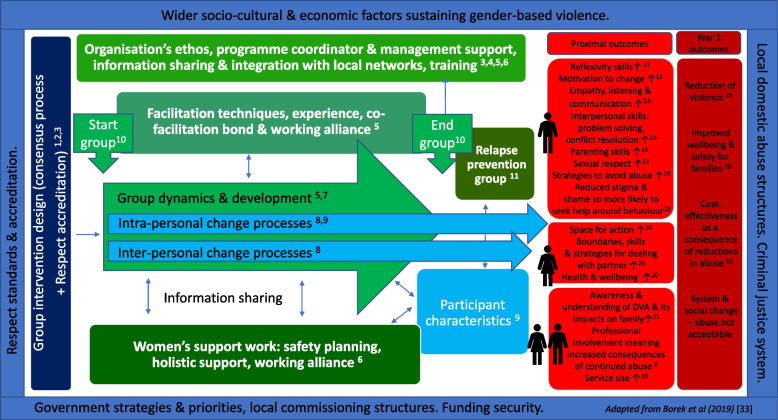
Theory of change [33, 34]

We undertook an evidence synthesis of existing domestic abuse perpetrator programmes to identify their key ingredients and limitations [18]. We used this to inform a modified two-stage Delphi consensus meeting with an invited range of DVA experts: representatives from Respect, practitioners from domestic violence services, the criminal justice system, general practice and academic researchers, the findings of which informed the group programme and manual [32]. A pilot trial confirmed it was feasible to recruit, randomise and retain male perpetrator and female victim/survivors [20]. Following the pilot, the main trial includes formal integration of the relapse prevention group (RPG) (discussed in more detail below), and additional emphasis on exercises to help with trauma. A theory of change was developed to summarise and capture key aspects of the intervention (Fig. 1).

#### Domestic abuse perpetrator programme

The DAPP intervention is a 23-week group programme with an initial full risk assessment and at least three additional one-to-one sessions. The two-and-a-half-hour weekly sessions are delivered by two DAPP facilitators (ideally, male/female dyads to model good gender role behaviours) from the local provider organisation. The programme runs as a rolling programme allowing new intakes of participants to join every 4 or 5 weeks. Intake weeks depend on the session focus, with certain sessions (such as *Sexual Respect*) being considered too difficult to be an introductory session for a new group member. The sessions are structured to follow the REPROVIDE DAPP manual whose elements include goal identification and goal setting, recognising abuse, denial and minimisation, intents of violence, basic anger management, identifying urges to perpetrate abuse and cooling down strategies, basic cognitive behavioural therapy, effects of DVA on partners and children, participant’s own childhood experiences, impacts on children, active listening, conflict resolution, masculinity, beliefs and expectations, sexual respect, attachment styles, building empathy, loving relationships, emotional abuse and accountability.

Individual sessions led by the DAPP coordinator are tailored to participants’ needs following initial and ongoing assessment with referrals and signposting to other services as needed, such as specialist support for substance abuse. Possible individual interventions may include deconstructing specific incidents of abuse or planning discussions with partners or children. To support quality assurance for the programmes, all our delivery partner organisations were already Respect-accredited or in the processes of accreditation.

#### Relapse prevention group (RPG)

Men who complete the 23-week programme will be able to access the RPG, also referred to as a *maintenance* group, if they wish. These meet monthly and are run by the local service provider team (facilitator or DAPP coordinator). These meetings are less structured than the programme, with an emphasis on *checking in* and how the participants are managing their behaviours. Men are offered the opportunity to attend the RPG for up to 6 months after the group programme ends.

#### Support and information sharing intervention for (ex)partners

The intervention for the female (ex)partners linked to men randomised into the intervention arm is the offer of one-to-one support from a women’s safety worker (WSW) for the duration of the group programme (irrespective of whether the man attends or completes the programme) and for up to 6 months after the programme ends, mirroring the men’s potential participation in the weekly groups and RPG. The WSW assesses risk and provides support and advocacy. The support is offered either face-to-face by telephone or online (see Supplementary Information: RESPONSE TO COVID-19 PANDEMIC). Contact is tailored to need. To manage risk for the (ex)partner and any children, the DAPP facilitators and the WSW regularly share information on an ongoing basis. In some cases, a male participant may have two (ex)partners receiving support. In these cases, where possible, a second WSW would support the second partner.

#### Usual care control arm

Men allocated to the usual care control arm will not receive any intervention or referrals from the research team. However, they are free to access any other services available to them. The research team may signpost the men to other appropriate services (for example mental health or substance abuse services) if appropriate. All women—regardless of their (ex)partner’s allocation—will be signposted to local and national women’s support services by a member of the research team dedicated to providing contact to the (ex)partners.

### Criteria for discontinuing or modifying allocated interventions {11b}

Participants may be excluded from the *intervention* post-recruitment if (i) they are disruptive towards the group, facilitators or DAPP coordinator; (ii) there is an increased risk of harm to the participant’s (ex)partner or children; (iii) they fail to attend three or more consecutive group sessions without agreement from the DAPP coordinator. Excluded participants will still be followed up unless the participant chooses to withdraw fully from the study. Participants may be excluded from the *trial* post-recruitment if there is an increased risk of harm to the participant’s (ex)partner or children or increased risk to a member of the research team.

### Strategies to improve adherence to interventions {11c}

Male participants are offered one-to-one sessions with programme coordinators where appropriate to discuss their motivation to continue with the intervention and to help them recognise the benefits of continuing. Female participants are offered the opportunity to simply have regular ‘check-in’ contact and can re-engage more fully with their safety worker if needed.

### Relevant concomitant care permitted or prohibited during the trial {11d}

There are no restrictions on participants accessing additional support relating to domestic abuse during the course of the trial after they have been recruited. In follow-up questionnaires, they will be asked to provide information relating to their use of mental health, therapeutic, social care, community and/or voluntary sector services.

### Provisions for post-trial care {30}

There are no plans for post-trial care. Follow-up of participants continues for 12 months post-recruitment. Services providing the intervention will be expected to refer participants to appropriate further services, depending on need.

### Outcomes {12}

#### Primary outcome measure

The primary outcome is male self-reported abuse based on the Abusive Behaviour Inventory (ABI) [35]. The original ABI consists of 30 questions; however, as recommended by Postmus et al. [36], we removed the item that asks male participants if they had ‘spanked’ their abused partner. Another item, which asks if male participants had told their partners that they are a ‘bad parent’, was amended to ‘bad person’, as not all participants were parents. The revised primary outcome measure of abuse therefore consists of 29 items and is hereafter referred to as the ABI-29.

#### Secondary outcomes: self-report measures

Secondary outcome measures for male participants and (ex)partners are given in Table 3. Those for (ex)partners include the ABI-R [36], physical and mental health status, substance and alcohol abuse and economic evaluation.

**Table 3:** Primary and secondary outcomes

**Questionnaires**	**Also known as / objectives:**	**Baseline**	**4 months**	**8 months**	**12 months**
**For male perpetrators and female victim/survivors**					
Socio-demographic measures	Age, number of children at home, ethnicity, income, occupation, sexuality.				
Resources use questions	Use of health and social services, criminal justice system, medication use, housing, employment and benefits, use of children’s services				
IMPACT [37]	IMPACT toolkit. To assess the effect of the intervention on measures of DVA				
IMPACT (selected) [37]	Selected questions from the IMPACT toolkit. To assess the effect of the intervention on measures of DVA				
EQ-5D-5L [38]	EuroQol European Quality of Life questionnaire (5-Dimensions 5-Level Version)				
SF-12 [39]	Short Form health questionnaire-12				
ICECAP-A [40]	ICEpop CAPability measure for Adults				
PHQ-9 [41, 42]	Patient Health Questionnaire -9. To assess the effect of the intervention on measures of health and wellbeing.				
GAD-7 [43, 44]	Generalised Anxiety Disorder assessment -7. To assess the effect of the intervention on measures of health and wellbeing.				
PC-PTSD [45]	Primary Care PTSD / Post-traumatic Stress Disorder scale. To assess the effect of the intervention on measures of health and wellbeing.				
AUDIT-C [46]	Alcohol Use Disorders Identification Test-C				
DUDIT [47]	Drug Use Disorders Identification Test				
Childhood experiences questions [48, 49]	Self-reported adverse childhood experiences (adapted from the PROVIDE survey and childhood sexual victimization				
Current or past physical &/or mental health problems	(self-reported), including treatment.				
Your children	Information about number of children and type and nature of contact – self report				
Your relationship	Relationship status, type and nature of contact and hopes for the future				
**For male participants only**					
**Primary Outcome Measure:** ABI-29 [35, 36]	Abusive Behaviour Inventory-29. To investigate the effectiveness of the group programme intervention on reducing men’s abusive behaviour against women.				
AQ-10 [50]	Autism spectrum Quotient				
IPVRAS- adapted [51]	Adapted Intimate partner violence responsibility attribution. To assess justifications and responsibility for DVA.				
PAS (Anger sub-scale) [52, 53]	Propensity for abusiveness Anger (affective ability) sub-scale. To assess the effect of the intervention.				
CPQ-SF [54]	Communications Patterns Short Form – adapted. To assess the effect of the perpetrator intervention on measures of health and wellbeing				
RFQ [55]	Reflective Functioning Questionnaire. To assess the effect of the perpetrator intervention on measures of health and wellbeing				
**For female participants only**					
ABI-R [36]	Revised Abusive Behaviour Inventory. To assess the effect of the intervention on measures of experience of DVA, health and wellbeing of female (ex)partners				
CHU-9D [56]	Proxy version: Child Health Utility Index – 9 Dimension. A health-related quality of life measure for one child (the child with the first birthday in the calendar year), which can be used to calculate QALYs (Quality Adjusted Life Years)				
Children’s resource use	Health care use and missed days at school for one child aged 5–18 years (the child whose birthday was first in the calendar year).				

#### Secondary outcomes: Police data

We will collect police-recorded DVA incidents and crimes perpetrated by our male participants. We will collect data for the 12-month period prior to randomisation and 12 months post-randomisation (see Supplementary Information: RESPONSE TO COVID-19 PANDEMIC). We will request for each male participant the following: (a) a count of the number of police incidents/crimes flagged as DVA; (b) the date(s) of the incident(s)/crimes; (c) the police case outcome for each incident/crime (for example, *No Further Action*, *Charge*, *Domestic Incident only*); (d) a count of the number of entries on the Log of Enquiries for each of these incidents; (e) risk scores/ratings for each of these incidents where available and whether referred to Multi Agency Risk Assessment Conference (MARAC).

Pre- and post-randomisation police DVA incident data are another measure of participant behaviour change. The risk scores/ratings and the police case outcome will also give an indication of the severity and nature of the abuse. Finally, the police data give an external source of data distinct from the participants’ self-reports of abuse.

#### Process evaluation

Programme process will be monitored to assess delivery of the intervention across the different service providers and to compare with the intended intervention model. Group attendance data and reasons for absence will be collected to help assess the intervention *dose* that each man received and their levels of engagement. Session notes from the group facilitators will be used in conjunction with video recordings of weekly sessions (which are part of normal DAPP delivery) and observations to ascertain the fidelity to the model (for example, were session objectives achieved, adherence to programme principles, adherence to or deviation from the manual). Numbers of one-to-one DAPP coordinator contact with men and the broad purpose for these individual sessions will be collected. The frequency and type of contact (face-to-face, online, telephone) and main purpose of contact between the WSW and (ex)partners will also be collected.

#### Nested qualitative study

A qualitative study will be undertaken using semi-structured interviews with study participants to elicit their perspectives on key aspects of the intervention and study experience. We will, for example, seek to gain feedback on experiences of the intervention (male and female intervention participants) and a better understanding of any changes in abusive behaviours during the study period (all male and female participants). Using topic guides, likely questions for male and female control participants will include whether they felt they were being monitored in any way while being part of the trial. We will interview some professional referrers and staff in different provider roles (DAPP coordinator, group facilitator, managers) regarding their experiences of referring to or delivering the group intervention and of supporting (ex)partners. Data will be collected on the context of the intervention at each centre partly through interviews and a few observations of the group sessions at each centre. Contextual data will also be captured through ongoing gathering of external information such as information about alternative DAPP providers in each centre and dates the COVID-19 lockdown rules changed (different for England and Wales). These qualitative data will help articulate likely challenges in future implementation (if the intervention proves cost-effective) and help to interpret the quantitative results. Interviews will be recorded on an encrypted device and stored securely on university servers. They will be transcribed and anonymised, and coded thematically by members of the research team, with some interviews being coded by more than one researcher to ensure inter-rater reliability.

#### Economic evaluation measures

All resource use in relation to participants’ own use of health services (secondary, community and mental health based), medications, personal social services (e.g. drug and alcohol team), voluntary sector services, criminal justice and legal services will be collected for the 12 months following randomisation by means of self-reported questionnaire, and in the case of police incidents, the police-recorded data. These questionnaires will also collect information on out-of-pocket expenses, e.g. personal counselling, complementary therapy, relocation costs and childcare costs, in addition to time off work.

Additionally, the use of health services and time off school by any children who primarily live with the supporting (ex)partner is being collected through their respective questionnaire. Three outcomes for the economic evaluation: EQ-5D-5L [38], SF-12 [39] and ICECAP-A [40] are being collected for all participants; additionally, the proxy version of the CHU-9D [56] is being collected in the supporting (ex)partner questionnaire for the child whose birthday is first in the calendar year. The NICE recommended mapping function [57] will be used to calculate quality-adjusted life years (QALYs) in relation to the EQ-5D-5L. The SF-6D and its preference weights [58] will be used to calculate QALYs in relation to the SF-12. A published value set [59] will be used to calculate QALYs in relation to the CHU-9D.

The resource use and costs incurred in relation to the intervention: the group intervention (both main and RPG) as well as the individual sessions for men and support from the WSW for the (ex)partners will be obtained from the budget breakdown.

### Participant timeline {13}

A potential participant will have an initial telephone discussion with a researcher for eligibility screening and an explanation of the study. If eligible and interested, a participant information sheet will be sent electronically or by post according to preference. If the potential participant is happy to proceed, a date and time will be agreed for a joint recruitment and assessment meeting with the potential participant, researcher and local DAPP coordinator. At the recruitment meeting, the researcher checks the potential participant’s ID, understanding of the study and re-checks eligibility, while the DAPP coordinator assesses suitability to potentially join a perpetrator group programme. If there are any concerns about lack of understanding, motivation or risk, the meeting may be paused, and another appointment made if more investigations are needed, or the potential participant requires more time to think. The process may be stopped altogether if it is felt inappropriate to recruit the potential participant or if he decides he no longer wishes to proceed. If the participant is happy to proceed, signed informed consent is taken by the researcher, and contact details of the participant’s (ex)partner[s] (up to two (ex)partners per male participant), general practice and any other agencies (mental health, substance misuse or social workers) will be requested.

The male participant will either complete the baseline questionnaire before the assessment (if online) or in the presence of the researcher if face-to-face (see also Supplementary Information: RESPONSE TO COVID-19 PANDEMIC). Once recruitment and baseline assessments are completed, the participant is randomised and immediately informed of the results and the next steps in the research (Table 4).

**Table 4:**
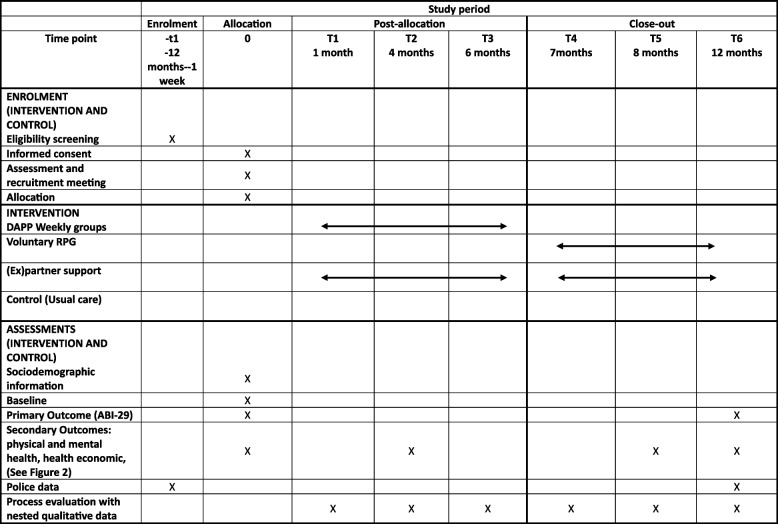
Schedule of enrolment, interventions and assessments

*DAPP* Domestic abuse perpetrator programme, *RPG* Relapse prevention group, *ABI-29* Abusive behaviour Inventory [29]

The female (ex)partners of perpetrator participants will be contacted about the study before the male assessment meeting where possible and the study explained to them. After the assessment, the (ex)partners will be informed of his participation and (for safety purposes) allocation in the study and invited to consider taking part in the study themselves. If potential study participation is agreed, a meeting with the researcher will be arranged at a safe and mutually convenient location or online or by phone. At this meeting, eligibility will be checked, informed consent sought and baseline questionnaire completed. (Ex)partners unable to meet in person may still participate in the study by returning electronic or postal consent and questionnaires (see Supplementary Information: RESPONSE TO COVID-19 PANDEMIC).

For (ex)partners of men allocated to the intervention arm, the associated women’s safety worker will contact the partner to offer support. This support will be offered whether or not the woman consents to participate in the study. (Ex)partners of men allocated to the control arm will be signposted to local and national DVA support services.

**Fig. 2:**
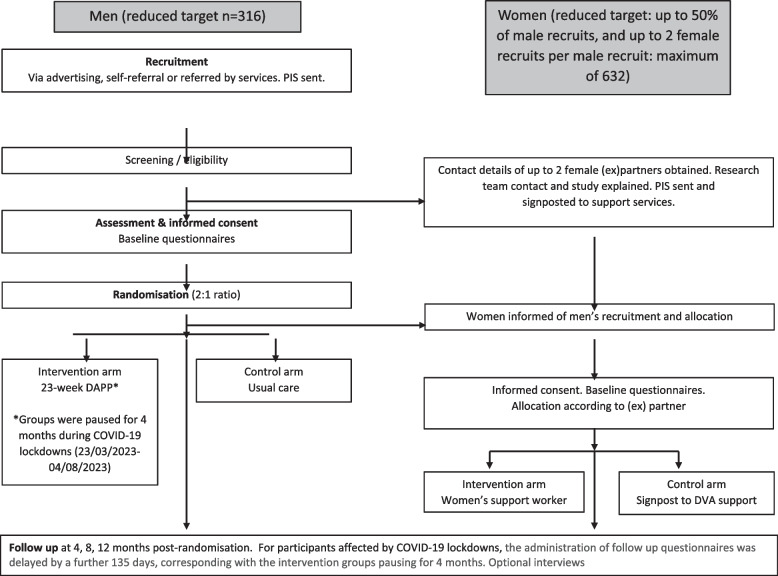
Participant timeline

The flow of male participants and recruited (ex)partners through the trial is summarised in Fig. 2 and in Table 4.

### Sample size {14}

In all power calculations, we have assumed a 2:1 allocation ratio (intervention: control) and applied a two-sided significance level of 5%. Our initial assumption was that a total of 219 participants would be available for analysis which, after inflation to allow for 40% attrition gives a total recruitment target of 366 participants (244 intervention and 122 control).

The (primary) power calculation used the power command in Stata 15.1, which yielded 79% power for an effect size of 0.4 standard deviations (0.4SD). While there is no established minimal clinically important difference on the ABI-29 scale, baseline data from male perpetrators in a group support study in the US using the ABI-30 found a standard deviation of 11 giving a detectable effect size corresponding to 4.4 points [60]. Such a difference is considered plausible and of a magnitude worthwhile detecting if the intervention had such an effect.

The design of the intervention allows a rolling intake of participants in the various groups; therefore, there is potential for clustering in that arm (with no such effects in the control arm). The degree of this effect (essentially one of additional inefficiency/compromise on power) will depend on the degree of clustering, the number of intervention groups and the extent to which these groups overlap as men enter and leave them. All of these are difficult to predict; the latter in particular means that a clear definition of a cluster is very likely to be elusive. Hence, we have not allowed for this in the above power calculation but have explored the potential for such clustering effects using the clsampsi command in Stata 15.1 [61]. Notwithstanding these uncertainties, with a (conservative) intra-cluster correlation coefficient (ICC) of 0.05, the above sample size would yield a power of 80% to detect a 0.435SD effect size. The originally planned sample size would therefore have about 80% power to estimate an effect size of between 0.4 to 0.435 SD, covering situations with zero/negligible clustering and for a conservative ICC.

### Reduction in sample size

Due to difficulties experienced in meeting the original target for recruitment, primarily from the first 18 months of recruitment being conducted during the COVID-19 pandemic, along with an extended recruitment period, a reduction of 50 in this target was subsequently approved by the Programme Steering Committee (PSC), Data Monitoring and Ethics Committee (DMEC), and the funding body. Using the SD of 10.7 derived from 201 baseline measurements in this trial and again assuming 40% attrition, the revised sample size of 316 to be recruited provides approximately 80% power to detect an effect size of 0.43SD under the primary assumption of no clustering. This slightly revised target difference, corresponding to 4.6 ABI-29 scale points (compared with the original of 4.4), was still deemed plausible and worth detecting to drive implementation of the programme after the trial.

### Recruitment {15}

The study will be advertised through posters and leaflets in general practices, pharmacists and other local community settings including sports and exercise venues, pubs and church halls. Local social services, DVA forums and national DVA networks and helplines (for example, Respect) have been made aware of the study through newsletters and presentations and will be able to refer men to the study. A prospective participant can also self-refer into the study. A digital social media campaign providing targeted Facebook and Google adverts to males using specific search terms (such as *anger management*) and further social media campaigns will make use of Twitter, Instagram and Facebook to direct potential referrers, such as social services, to the study. Regular presentations and discussions will be held with these potential referrers to ensure they fully understand the rationale for the trial and the processes for men who are referred and for their (ex)partners.

## Assignment of interventions: allocation

### Sequence generation {16a}

Computer-generated sequence using a minimisation approach, stratified by centre. Each participant will be allocated to the group that would result in the smallest imbalance in the variable ‘partner status’ with probability of 0.8 to avoid the allocations becoming predictable.

### Concealment mechanism {16b}

Computer system accessed remotely by researcher. The male participant will be registered with the study before allocation is revealed.

### Implementation {16c}

The recruiting researcher accesses a website remotely, enters the required details and is immediately informed of the randomisation allocation. This is shared straightaway with the participant and the service provider.

## Assignment of interventions: blinding

### Who will be blinded? {17a}

Once participants are recruited and randomised, it is not possible to mask participants to their allocation. For the purposes of safety, it would not be appropriate to mask a participant’s (ex)partners, local intervention delivery team or local WSW to the participant’s allocation. Members of the research study team need to be unblinded to carry out research activities. However, all data entry and data quality checks will be performed by study administrative staff, masked to trial allocation.

### Procedure for unblinding if needed {17b}

Not applicable, given that it is not appropriate for the research team, intervention delivery teams, or male or female participants to the male participant allocation.

## Data collection and management

### Plans for assessment and collection of outcomes {18a}

Data will be collected at four timepoints: baseline, then 4, 8 and 12 months after recruitment. The primary method of data collection is by self-reported questionnaire. Participants will be asked to complete the questionnaires via an online survey (via a unique Research Electronic Data Capture (REDcap) weblink hosted by the university of Bristol) [62, 63], on paper in a face-to-face meeting, or by telephone with a researcher, according to preference or need.

Figure 2 sets out the details of the outcome measures being collected at which time points and the objectives of these measures.

Measures only collected at baseline are as follows: sociodemographic variables (age, ethnicity, sexuality, religion, education, employment, income, housing and number of children at home); current self-reported physical disability or mental health problems, including treatment; autism spectrum quotient (AQ-10) [50]; adverse childhood experiences (adapted from the PROVIDE survey [48] and juvenile victimisation questionnaire [41]) and the IMPACT monitoring toolkit (client or partner T0) [37].

### Plans to promote retention and complete follow-ups {18b}

The research team will endeavour to maintain contact and trial engagement with all participants every 6–8 weeks via text messaging (according to contact preference). Two to three weeks prior to a questionnaire’s due date, participants will be alerted by the method of their choice (text, email, or phone call) to the forthcoming questionnaire. Up to seven reminders may be sent via different means (calls, text, email) if the follow-up questionnaire has not been completed.

An eligible potential participant who chooses not to participate is counted as a *decline* and reasons for declining will be collected. Once consented, a participant may withdraw at any time should they wish. Participants allocated to the intervention who no longer wish to be involved in the programme will still be followed up with questionnaires. No further follow-up data will be requested from any individuals who explicitly wish to fully withdraw from the trial, but data collected up to that point will be retained and included for data analysis.

### Data management {19}

REDCap is a secure, web-based electronic data capture (EDC) system designed for the collection and management of research (as distinct from administrative) data. Although the REDCap system has been developed (and it is supported) by Vanderbilt University, Bristol Medical School has set up its own infrastructure to host the REDCap application so that all elements reside within UoB.

All questionnaire data are stored in a secured UoB server subject to standard UoB security procedures. Outcome data are anonymised by use of the ID number and no personal details will be held on the outcome database.

Both outcome (REDCap) and Administrative (AdminDB) data are secured using robust security mechanisms. Both systems also have audit logs cataloguing individual changes with data/time, old value, new value and the identity of the user who made the change.

### Confidentiality {27}

All personal information and research data can only be accessed by authorised accounts and authorisation can only be granted by specific people such as the chief investigator, the programme manager, or members of the research team.

Administrative data including names, addresses, contact details and other personalised data will be stored on a bespoke management database (AdminDB), designed and managed by the Bristol Trials Centre (BTC) and held in the University of Bristol (UoB) SQL Server Cluster. All participants will be allocated a unique numerical ID number which will be used to provide anonymity and track their data.

Where possible, personal identifiable details will be removed from hard-copy documents and replaced with the participant’s unique trial identification number. During the study, all hard-copy documents containing patient identifiable data will be stored separately from research data in (as a minimum) locked filing cabinets within alarmed, access-restricted University buildings of each of the research centres. Only local research teams will have access to these locked cabinets.

Electronic data will only be accessible via a password-protected database held on a secure server.

### Plans for collection, laboratory evaluation and storage of biological specimens for genetic or molecular analysis in this trial/future use {33}

N/A. No biological specimens will be collected in this study.

## Statistical methods

### Statistical methods for primary and secondary outcomes {20a}

Descriptive statistics will be used to summarise all baseline characteristics and outcome measures in both study arms.

The primary outcome of the ABI-29 will be analysed using a linear regression model that will include the baseline ABI-29 score, the stratification factor of centre and minimisation factor of relationship status. The primary analysis will be performed on a complete case basis and according to the arm to which the participant was allocated. Sensitivity analyses will be conducted to explore the effects of the number of sessions attended and (if possible, given the eventual structure of the intervention groups) any effects of clustering. Secondary outcomes will be analysed using linear or logistic regression models as appropriate. If found, substantial imbalances of variables at baseline (determined using descriptive statistics) will be explored in sensitivity analyses.

### Interim analyses {21b}

Not applicable unless requested by the DMEC.

### Methods for additional analyses (e.g. subgroup analyses) {20b}

Further secondary analyses will include pre-specified subgroup analyses according to age and to the two stratification/minimisation variables, investigated by introducing the relevant interaction with treatment allocation into the regression model. In addition, to assess the stability of any intervention effect, we will fit a mixed model for the primary outcome(s) at 4, 8 and 12 months, adjusted for baseline measures. We will also investigate obtaining complier-average causal effect (CACE) estimates using instrumental variable techniques to investigate the efficacy of the intervention while accounting for degree of adherence to the programme [64].

A statistical analysis plan will be produced by the trial team and agreed with the PSC and DMEC before the commencement of any comparative analyses. It will be posted on PURE, UoB’s research information system and repository of scholarly works: http://www.bristol.ac.uk/red/research-policy/pure/.

### Methods in analysis to handle protocol non-adherence and any statistical methods to handle missing data {20c}

Intention-to-treat analysis will be carried out. All randomised participants, including those who drop out of or are excluded from the intervention will be included in the analysis. Sensitivity analyses will be conducted to explore the effects of missing data.

#### Economic evaluation

The economic evaluation over a 1-year time horizon will be conducted from three perspectives: health and social care, public sector and society will employ a cost-consequences framework such that costs are reported alongside the economic outcomes (QALYs (derived from the SF-12 [38], EQ-5D-5L [37] and CHU-9D [40]) and ICECAP-A [39] as well as the primary and other secondary outcomes.

The resource use will be valued using the most up to date reference costs where possible; otherwise, estimates from literature and those reported by participants will be used, and if necessary, adjusted for inflation [65, 66].

Resource use and costs will be grouped by the categories: health and social care, public sector, participants, society. Within these categories, the resource use, and costs to the men, supporting (ex) partners and children will be reported separately.

Descriptive statistics will be used to summarise resource use, costs and outcome measures by study arm.

Appropriate regression analysis will be used to analyse the costs and outcomes. Costs will be adjusted for the baseline ABI-29 score, the stratification factor of centre and minimisation factor of relationship status, additionally the QALYs will be adjusted for the baseline utility score [67].

Sensitivity analyses will be used to explore the effects of methodological assumptions as well as those of missing data.

#### Process evaluation

The aim of the process evaluation is to assess the implementation of the intervention, explore the contexts where the intervention was effective (or not) and examine the experiences and acceptability of those delivering and participating in the study.

Quantitative elements of the process evaluation (attendance, number of contacts and completion rates) will be summarised by descriptive statistics. A nested qualitative study will analyse transcribed interviews supplemented with video recordings, observations and field notes. A coding framework will be developed, and data sets analysed thematically with triangulation from different data sources. PPI groups will be consulted about emerging themes.

### Plans to give access to the full protocol, participant-level data and statistical code {31c}

The full protocol will be available upon reasonable request. Participant-level data and statistical code will be accessible through the University of Bristol Research Data Repository.

## Oversight and monitoring

### Composition of the coordinating centre and trial management committee {5d}

This RCT is one workstream within a wider NIHR Programme Grants for Applied Research programme of research: *Reaching Everyone Programme of Research On Violence in diverse Domestic Environments* (REPROVIDE). A management committee, including all co-investigators manages the programme, including this trial. A trial management group (TMG) consists of the chief investigator, principal investigators of each workstream, programme manager, statisticians, health economists and researchers. A programme executive committee (EC) consisting of academics, service providers and trial collaborators also provides guidance and oversight.

Strategic and governance oversight is provided by an independent programme steering committee (PSC).

### Composition of the data monitoring committee, its role and reporting structure {21a}

An independent DMEC reviews study progress and safety data as provided by the TMG, and any ethical issues in keeping with the DMEC charter and reports to the PSC. The PSC has the authority to stop the trial should there be significant safety issues or cause for concern.

As previously mentioned, there are two PPI groups for the research. A group of female victim/survivors who had experienced DVA and a separate group of men who had attended and completed a DAPP (either the REPROVIDE DAPP or another DAPP). Initially, the men’s PPI group also provided an opportunity for men to attend an RPG in a supportive environment order to reflect on the challenges to maintaining positive behaviour change. The men are not paid for their time, but a meeting venue is provided. Halfway through the trial this double function stopped, and the men’s PPI group was reformed in a similar way to the women’s PPI group with all PPI members being invited to take part solely for the purpose of supporting the research and paid as consultants for their time and expertise. Both PPI groups are consulted two–three times per year on the design of the research and the intervention, including questionnaire content and which validated quality of life measure to use, recruitment materials and terminology. Two PPI members are also invited to attend the PSCs and ECs.

### Adverse event reporting and harms {22}

Given the complex nature of this participant population, there is a high risk to the safety of the participant, their (ex)partner(s), any children they have contact with, and to a lesser extent, to the facilitators delivering the intervention and to the researchers. Where possible, female (ex)partners will be notified of their male partner’s potential participation in the study and after (male) recruitment, all female (ex)partners will be contacted again about allocation, to check that they received that communication, offered signposting and invited to take part in the study.

Safeguarding of the (ex)partner and any associated children are of paramount importance; indeed, (ex)partners of participants in the intervention arm will be offered support from the WSW regardless of whether they wish to take part in the study. The WSW will communicate closely with the local DAPP coordinator/facilitators to be aware of any changes in risk while the male participants are undertaking the programme.

All partner organisations will be alert to and report any serious incidents to the research team where they have been made aware of hospitalisation of study participants, or incidents involving serious violence which they feel the research team should be made aware of. This may be through direct disclosure from participants themselves, or through indirect means—for example, regular police reports or local court proceedings. Health-related incidents will follow conventional adverse event (AE) and serious adverse event (SAE) reporting procedures. All SAEs will be assessed for intensity, causality and expectedness by the programme clinician and reported to the sponsor, study oversight committees and Research Ethics for monitoring and review as necessary.

### Frequency and plans for auditing trial conduct {23}

The DMEC and PSC both meet twice a year and review the conduct of the trial. The DMEC and PSC meetings are linked and in a sequence, so that any concerns raised in the DMEC are passed to the PSC. Examples of issues raised in the DMEC are the routine monitoring and checking of returned questionnaires for risk and safeguarding purposes (July 2022) and a review of serious adverse events and adverse events (March 2023).

### Plans for communicating important protocol amendments to relevant parties (e.g. trial participants, ethical committees) {25}

Any substantial amendments to the trial protocol will require approval of the South Central Oxford B NHS Research Ethics Committee. Minor amendments will be notified to and recorded by the study sponsor, the University of Bristol. Recruitment material and the patient information sheets will be updated if amendments involve changes to eligibility criteria.

### Dissemination plans {31a}

A publication plan will be drafted as part of a wider dissemination and knowledge mobilisation strategy. Findings will be disseminated via final report and peer-reviewed publications, as well as guidance and briefing reports for professionals, commissioners and policymakers.

Participants who have asked to be kept informed will be provided with the findings and we will take advice from our PPI representatives as to how best to provide this in accessible formats.

## Discussion

The fully powered trial will provide robust evidence of the effectiveness and costs of a domestic abuse perpetrator programme. The intervention and study design are built on a broad evidence base, with input and consultation from experts, stakeholders and PPI members and with particular emphasis on the safety and health outcomes of the female victim/survivor partners and ex-partners.

The embedded process evaluation will provide further insights in the mechanisms and barriers, experiences and contexts of participants and their journey through a perpetrator programme. Economic evaluations have been absent in prior studies of perpetrator programmes. Therefore, this study seeks to address this omission and will benefit from the use of broader perspectives.

## Trial status

The current protocol is version 8, 22nd September 2022. Recruitment began on the 21st Oct 2019, but was somewhat delayed as a result of COVID-19. Approximate date when recruitment will be completed for male participants: June 2023, and for female participants, July 2023. Follow-up of participants will continue for another 12 months, until June/July 2024.

### Supplementary Information

**Additional file 1.** RESPONSE TO COVID-19 PANDEMIC.

## Data Availability

The quantitative datasets generated and analysed during the study will be available to bona fide researchers on reasonable request at the University of Bristol Research Data repository. The qualitative and police datasets generated and analysed during the study will not be publicly available due to the highly sensitive nature of the data which might place victims/survivors of domestic abuse at further risk.

## Abbreviations

BNSSG: Bristol, North Somerset and South Gloucestershire
DAPP: Domestic abuse perpetrator programme
DVA: Domestic violence and abuse
NICE: National Institute for Health and Care Excellence
PPI: Patient and public involvement
RPG: Relapse prevention group
WSW: Women’s safety worker

## References

[1] Campbell J, Jones AS, Dienemann J (2002). Intimate partner violence and physical health consequences. Arch Intern Med.

[2] Loxton D, Dolja-Gore X, Anderson AE, Townsend N (2017). Intimate partner violence adversely impacts health over 16 years and across generations: a longitudinal cohort study. PLoS One.

[3] Trevillion K, Oram S, Feder G, Howard LM (2012). Experiences of domestic violence and mental disorders: a systematic review and meta-analysis. PLoS One.

[4] Chandan JS, Thomas T, Bradbury-Jones C, Russell R, Bandyopadhyay S, Nirantharakumar K (2020). Female survivors of intimate partner violence and risk of depression, anxiety and serious mental illness. Br J Psychiatry.

[5] Office for National Statistics. Domestic abuse in England and Wales overview: November 2022: statistical bulletin: ONS; 2022. Available from: https://www.ons.gov.uk/peoplepopulationandcommunity/crimeandjustice/bulletins/domesticabuseinenglandandwalesoverview/november2022.

[6] Walby S, Towers J, Francis B (2016). Is violent crime increasing or decreasing? A new methodology to measure repeat attacks making visible the significance of gender and domestic relations. Brit J Criminol.

[7] Oliver R, Alexander B, Roe S, Wlasny M. The economic and social costs of domestic abuse. Home Office (UK) Report no. 107. 2019. https://assets.publishing.service.gov.uk/government/uploads/system/uploads/attachment_data/file/918897/horr107.pdf.

[8] Karakurt G, Koç E, Çetinsaya EE, Ayluçtarhan Z, Bolen S (2019). Meta-analysis and systematic review for the treatment of perpetrators of intimate partner violence. Neurosci Biobehav Rev.

[9] NICE (National Institute for Health and Care Excellence). Public health guideline on Domestic violence and abuse: multi-agency working (PH50). 2014. https://www.nice.org.uk/guidance/ph50.

[10] Respect. The respect standard. 3rd edition. 2017. http://empathygap.uk/Respect_Perpetrators%20Accreditation%20Standard_2017.pdf.

[11] Akoensi TD, Koehler JA, Lösel F, Humphreys DK (2013). Domestic violence perpetrator programs in Europe, part II: a systematic review of the state of evidence. Int J Offender Ther Comp Criminol.

[12] Arias E, Arce R, Vilariño M (2013). Batterer intervention programmes: a meta-analytic review of effectiveness. Psychosoc Interv.

[13] Smedslund G, Dalsbø TK, Steiro A, Winsvold A, Clench‐Aas J. Cognitive behavioural therapy for men who physically abuse their female partner. Cochrane Database of Systematic Reviews 2011, Issue 2. Art. No.: CD006048. 10.1002/14651858.CD006048.pub2. 10.1002/14651858.CD006048.pub2 PMC12047670 17636823

[14] Vigurs CA, Schucan Bird K, Quy K, Gough D (2016). The impact of domestic violence perpetrator programmes on victim and criminal justice outcomes: a systematic review of reviews of research evidence.

[15] Gondolf EW (1999). A comparison of four batterer intervention systems: do court referral, program length, and services matter?. J Interpers Violence.

[16] Gondolf EW (2012). Future of batterer programs: reassessing evidence-based practice.

[17] Lilley-Walker SJ, Hester M, Turner W (2018). Evaluation of European domestic violence perpetrator programmes: toward a model for designing and reporting evaluations related to perpetrator treatment interventions. Int J Offender Ther Comp Criminol.

[18] Turner W, Morgan K, Hester M, Feder G, Cramer H (2003). Methodological challenges in group-based randomised controlled trials for intimate partner violence perpetrators: a meta-summary. Psychosoc Interv.

[19] Kelly L, Westmarland N. Domestic violence perpetrator programmes: Steps towards change. Project Mirabal final report. Durham: Durham University. 2015. https://projectmirabal.co.uk/wp-content/uploads/2020/06/ProjectMirabalfinalreport.pdf.

[20] Cramer H, Gaunt DM, Shallcross R, Bates L, Kandiyali R, Sardinha L, et al. Randomised pilot and feasibility trial of a group intervention for men who perpetrate intimate partner violence against women. 2023. 10.21203/rs.3.rs-2543341/v1. Preprint available at: randomised pilot and feasibility trial of a group intervention for men who perpetrate intimate partner violence against women - Abstract - Europe PMC.

[21] Phillips R, Kelly L, Westmarland N (2013). Domestic violence perpetrator programmes: an historical overview: discussion paper.

[22] Crowell NA, Burgess AW (1996). Understanding violence against women.

[23] Rees A, Rivett M (2005). ‘Let a hundred flowers bloom, let a hundred schools of thought contend’: towards a variety in programmes for perpetrators of domestic violence. Probat J.

[24] Yalom ID, Leszcz MC (2005). The theory and practice of group psychotherapy.

[25] Barner JR, Carney MM (2011). Interventions for intimate partner violence: a historical review. J Fam Viol.

[26] Pence E, Paymar M (1993). Education groups for men who batter: the Duluth model.

[27] Räsänen E, Holma J, Seikkula J (2012). Dialogical views on partner abuser treatment: balancing confrontation and support. J Fam Violence.

[28] O’Connor A, Morris H, Panayiotidis A, Cooke V, Skouteris H (2021). Rapid review of men’s behavior change program. Trauma Violence Abuse.

[29] Bates EA, Graham-Kevan N, Bolam LT, Thornton A (2017). A review of domestic violence perpetrator programs in the United Kingdom. Partn Abus.

[30] Craig P, Dieppe P, Macintyre S, Michie S, Nazareth I, Petticrew M. Developing and evaluating complex interventions: the new Medical Research Council guidance. BMJ. 2008;337a1655. 10.1136/bmj.a1655. 10.1136/bmj.a1655 PMC2769032 18824488

[31] Skivington K, Matthews L, Simpson SA, Craig P, Baird J, Blazeby JM (2021). A new framework for developing and evaluating complex interventions: update of Medical Research Council guidance. BMJ.

[32] Morgan K, Cramer H, Feder G. REPROVIDE Development: a modified Delphi consensus process. Briefing Note 1. 2023. Available from https://www.bristol.ac.uk/media-library/sites/primaryhealthcare/documents/reprovide/Briefing%20note%201%20-%20REPROVIDE%20Development%20-%20A%20modified%20Delphi%20consensus%20process.pdf.

[33] Borek AJ, Abraham C, Greaves CJ, Gillison F, Tarrant M, Morgan-Trimmer S (2019). Identifying change processes in group-based health behaviour-change interventions: development of the mechanisms of action in group-based interventions (MAGI) framework. Health Psychol Rev.

[34] Horvath AO, Greenberg LS (1989). Development and validation of the Working Alliance Inventory. J Couns Psychol.

[35] Shepard M, Campbell JA (1992). The Abusive Behavior Inventory: a measure of psychological and physical abuse. J Interpers Violence.

[36] Postmus JL, Stylianou AM, McMahon S (2016). The abusive behavior inventory-revised. J Interpers Violence.

[37] Work with Perpetrators European Network (WWP-EN). Impact monitoring toolkit. 2015. Available from: https://www.work-with-perpetrators.eu/impact.

[38] Herdman M, Gudex C, Lloyd A, Janssen MF, Kind P, Parkin D (2011). Development and preliminary testing of the new five-level version of EQ-5D (EQ-5D-5L). Qual Life Res.

[39] Ware J, Kosinski M, Keller SD (1996). A 12-Item Short-Form Health Survey: construction of scales and preliminary tests of reliability and validity. Med Care.

[40] Al-Janabi H, Flynn TN, Coast J (2012). Development of a self-report measure of capability wellbeing for adults: the ICECAP-A. Qual Life Res.

[41] Kroenke K, Spitzer RL, Williams JB (2001). The PHQ-9: validity of a brief depression severity measure. J Gen Intern Med.

[42] Manea L, Gilbody S, McMillan D (2012). Optimal cut-off score for diagnosing depression with the Patient Health Questionnaire (PHQ-9): a meta-analysis. Can Med Assoc J.

[43] Spitzer RL, Kroenke K, Williams JB, Löwe B (2006). A brief measure for assessing generalized anxiety disorder: the GAD-7. Arch Intern Med.

[44] Kertz S, Bigda-Peyton J, Bjorgvinsson T (2013). Validity of the Generalized Anxiety Disorder-7 scale in an acute psychiatric sample. Clin Psychol Psychother.

[45] Prins A, Bovin MJ, Smolenski DJ, Marx BP, Kimerling R, Jenkins-Guarnieri MA (2016). The Primary Care PTSD Screen for DSM-5 (PC-PTSD-5): development and evaluation within a veteran primary care sample. J Gen Intern Med.

[46] Saunders JB, Aasland OG, Babor TF, Delafuente JR, Grant M (1993). Development of the alcohol-use disorders identification test (AUDIT) - Who collaborative project on early detection of persons with harmful alcohol-consumption .2. Addiction.

[47] Berman AH, Bergman H, Palmstierna T, Schlyter F (2005). Evaluation of the Drug Use Disorders Identification Test (DUDIT) in criminal justice and detoxification settings and in a Swedish population sample. Eur Addict Res.

[48] Hester M, Ferrari G, Jones SK, Williamson E, Bacchus LJ, Peters TJ (2015). Occurrence and impact of negative behaviour, including domestic violence and abuse, in men attending UK primary care health clinics: a cross-sectional survey. BMJ Open.

[49] Yakubovich AR, Heron J, Feder G, Fraser A, Humphreys DK (2019). Intimate partner violence victimisation in early adulthood: psychometric properties of a new measure and gender differences in the Avon Longitudinal Study of Parents and Children. BMJ Open.

[50] Allison C, Auyeung B, Baron-Cohen S (2012). Toward brief “red flags” for autism screening: the short autism spectrum quotient and the short quantitative checklist in 1,000 cases and 3,000 controls. J Am Acad Child Adolsc Psychiatry.

[51] Lila M, Oliver A, Catala-Minana A, Galiana L, Gracia E (2014). The intimate partner violence responsibility attribution scale (IPVRAS). Eur J Psychol Appl L.

[52] Dutton DG (1995). A scale for measuring propensity for abusiveness. J Fam Violence.

[53] Dutton DG, Landolt MA, Starzomski A, Bodnarchuk M (2001). Validation of the propensity for abusiveness scale in diverse male populations. J Fam Violence.

[54] Christensen A, Sullaway M. The communication patterns questionnaire. 1984.

[55] Fonagy P, Luyten P, Moulton-Perkins A, Lee Y-W, Warren F, Howard S (2016). Development and validation of a self-report measure of mentalizing: the reflective functioning questionnaire. PLoS One.

[56] Stevens K (2009). Developing a descriptive system for a new preference-based measure of health-related quality of life for children. Qual Life Res.

[57] Hernandez-Alava M, Pudney S (2017). Econometric modelling of multiple self-reports of health states: the switch from EQ-5D-3L to EQ-5D-51, in evaluating drug therapies for rheumatoid arthritis. J Health Econ.

[58] Brazier J, Roberts J, Deverill M (2002). The estimation of a preference-based measure of health from the SF-36. J Health Econ.

[59] Stevens K (2012). Valuation of the child health utility 9D index. Pharmacoeconomics.

[60] Petrik ND, Gildersleevehigh L, Mcellistrem JE, Subotnik LS (1994). The reduction of male abusiveness as a result of treatment - reality or myth. J Fam Violence.

[61] Batistatou E, Roberts C, Roberts S (2014). Sample size and power calculations for trials and quasi-experimental studies with clustering. Stand Genomic Sci.

[62] Harris PA, Taylor R, Thielke R, Payne J, Gonzalez N, Conde JG (2009). Research electronic data capture (REDCap)–a metadata-driven methodology and workflow process for providing translational research informatics support. J Biomed Inform.

[63] Harris PA, Taylor R, Minor BL, Elliott V, Fernandez M, O’Neal L (2019). The REDCap consortium: building an international community of software platform partners. J Biomed Inform.

[64] Dunn G, Emsley R, Liu H, Landau S, Green J, White I (2015). Evaluation and validation of social and psychological markers in randomised trials of complex interventions in mental health: a methodological research programme. Health Technol Assess.

[65] Personal Social Sciences Research Unit (PSRU). Unit Costs of Health and Social Care programme (2022 – 2027). https://www.pssru.ac.uk/unitcostsreport/. Accessed 16 May 2023.

[66] NHS England. National cost collection for the NHS undated. Available from: https://www.england.nhs.uk/costing-in-the-nhs/national-cost-collection/.

[67] Manca A, Hawkins N, Sculpher MJ (2005). Estimating mean QALYs in trial-based cost-effectiveness analysis: the importance of controlling for baseline utility. Health Econ.

