# Supplementary material for: The effectiveness and cost-effectiveness of a group domestic abuse perpetrator programme: protocol for a randomised controlled trial
Source: Trials. 2023 Sep 28;24:617. doi: 10.1186/s13063-023-07612-6 (PMC10540403; doi:10.1186/s13063-023-07612-6)
Supplement: Supplementary file 1 — Additional file 1. RESPONSE TO COVID-19 PANDEMIC. [file 13063_2023_7612_MOESM1_ESM.docx]

## Additional file 1: RESPONSE TO COVID-19 PANDEMIC

**COVID-19 background**

In December 2019, China alerted the World Health Organisation (WHO) to a number of cases of a type of flu in Wuhan, Hubei province. Despite efforts to contain the virus, by 30th January 2020 it was declared a Public Health Emergency of International Concern and on the 11^th^ March 2020 COVID-19 was characterised as a pandemic by the WHO. As a result of this and once the virus had reached a critical point within the UK, a range of mitigation measures have been advised by the UK’s National Health Service, the Department of Health and Social Care, the National Institute for Health Research, and by the UK government. These measures include ‘social distancing’ which involve: avoiding contact with anyone displaying symptoms of COVID-19; avoiding non-essential public transport; working from home where possible; and avoiding large gatherings (including family gatherings) and small public spaces such as pubs and restaurants. On 23rd of March 2020, throughout the UK, this was tightened further into a formal lock-down, with all non-essential business closed, further isolating people in their homes.

## Implications of COVID-19 for the REPROVIDE Trial

The implications for the REPROVIDE randomised controlled trial and for potential participants are profound. Early indications from China are that the incidence of DVA increased when self-isolation was imposed and services are concerned that a similar increase will happen in the UK as a result of families having to self-isolate, compounded by increasing uncertainty over jobs and financial security for families (Women’s Aid, 2020). In this context, the victim/survivor support and signposting services to (ex)partners, and the identification and monitoring of abusive men that are provided within the context of the REPROVIDE study are vitally important services to continue. This we plan to do in a number of ways.

## Participants already recruited to REPROVIDE

We define male participants disrupted by COVID-19 as anyone randomised (the index date) before or on 30^th^ June 2020. We define female participants disrupted by COVID-19 as anyone recruited and linked to a man who was randomised before or on 30^th^ June 2020. For those already recruited, we will:

1. Send an initial update to all participants (by post or email, as preferred) confirming their continued participation in the trial. Updates are in the current protocol (v.8 22^nd^ March 2022);
2. For those in the intervention arm, inform them that the weekly DAPP groups have been suspended for men. However, the regular contact from the DAPP coordinator will continue. While regular contact is as per protocol, the suspension of groups is a change to the protocol in response to COVID-19;
3. Send a monthly check-in text with reminders of who to contact when needed, as per protocol;
4. Remind all participants to continue to expect 4-, 8-, 12-month questionnaires, as per protocol;
5. In the update to female (ex)partners (both intervention and control) we will include a list of local and national domestic abuse services.

In continuing to follow-up participants who have already been recruited, we will be able to measure both men’s self-reports of abusive behaviour and mental health throughout this crisis and reports of abusive behaviours from recruited (ex)partners. We will continue to monitor all contact with participants for safeguarding concerns and report those concerns and serious/adverse events as normal. Research team members will continue to check returned postal questionnaires, reviewing these for safeguarding concerns.

## Intervention arm: Weekly group domestic abuse perpetrator programme and support to linked (ex)partners via the women’s safety worker

As a result of the advice (and subsequent government order across the UK) to impose social distancing, the decision has been made to suspend all our group perpetrator programmes in all four sites with effect from 17th March 2020 until 4^th^ August 2020. Delivery partners will, however, maintain regular contact through weekly or two-weekly phone-calls or texts (or similar contact by another method) with male participants in the intervention arm. This has been termed ‘holding’ and for intervention men will be in the form of advice and support. An agreement has been reached with our partners on what ‘holding’ constitutes and this will be reviewed regularly. The DAPP coordinator will react appropriately to crisis situations and carry out safeguarding activities as and when the men share this information. Tools such as ‘Time Out’ will be used where appropriate, such as to de-escalate tensions, but the DAPP coordinators will not seek to proactively guide men to unpick their abusive patterns as they would in an individually delivered behaviour change programme. The (ex)partners of the intervention men will still be able to contact their women’s safety worker as usual.

The research team have developed templates for recording all male and female contacts and will collect this data from delivery partners on a regular basis to ensure accurate descriptions of the intervention alterations needed during COVID-19. A buddying system will be in place for the women’s safety workers in two of the sites in case of illness during the COVID-19 period with the other two sites having more extensive backup cover for victim/survivor support within their own organisations. The research team and all delivery partners will continue to engage in regular (virtual) meetings to troubleshoot concerns, to monitor how the one-to-one ‘holding’ contact for men progresses and to increase the consistency in this contact between sites.

## Continued (virtual) recruitment to the trial

We will continue to accept referrals into the study and the research team will go through the recruitment process as outlined in the protocol and will inform potential participants that we remain open to recruitment. In the light of COVID-19 restrictions, an addition to the above referral and recruitment procedure is that the research team will contact the ex/partner at this point, prior to proceeding to recruitment of the man, in order to get the ex/partner’s views on how safe she and her family would feel if he was involved in the study, and particularly if he was assigned the control arm group. It will be particularly important to check this if a couple are in isolation in the same household. As long as the ex/partner is satisfied his involvement in the study will not increase the level of risk to her and her family, then recruitment will proceed. For as long as face to face meetings cannot take place due to Government restrictions, assessments will take place virtually, using either Skype, WhatsApp or telephone.

Consent from the participant will be taken verbally, with the researcher talking him through each item on the consent form, initialling each item and signing the form on the participants’ behalf. This verbal consent process will be audio recorded where possible. A copy of the consent form with the method of consent agreement stated will be posted or emailed to the participant for his records. Although these options for informed consent are not as rigorous and are more open to bias compared with ‘normal’ trial proceedings, we feel that the ethical imperative to continue to identify and recruit abusive men and their ex/partners into the study reasonably balances these potential dangers.

At the beginning of any contact call, some additional risk management questions will be asked to ensure that, at the present time, the participant is in an environment which is both safe and private. Some basic questions on current circumstances may be added.

The participant will be asked to compete the baseline questionnaire either by a link to completing it immediately online, or by going through the questions with the researcher. If a participant is allocated to the intervention group, the next steps are explained to him, and the DAPP coordinator will arrange a further one-to-one meeting (which includes a full risk assessment) which will take place over phone/Skype/WhatsApp or similar. The DAPP coordinator will then remain in contact with the participant on a regular basis until the group programme is able to re-start. If a participant is allocated to the control arm the next steps are explained to him. All participants will be given a timeline of when to expect reminder contact and questionnaires for 4, 8 and 12 months. Recruitment of (ex)partners will be carried out as outlined in sections S.6 to S.9 (protocol v.8). However, all (ex)partners ex/partner recruitment will take place over the phone or online via Skype/WhatsApp or by email unless face-to-face is specifically preferred by the (ex)partner and it is safe to conduct for all parties.

## COVID-19 specific qualitative work enhanced and expanded

The nested qualitative study will be expanded to include questions in the topic guides relating to the impact of the pandemic on the male and female participants. Questions will added to assess whether and how abusive behaviour may have increased (or decreased) as a result of self-isolation. With regards to sampling, because the recruited numbers of participants are still low, we will invite all participants recruited to date to be involved (subject to the Trial Safety Protocols).

Male and female participants in both the intervention and control arms will be asked to take part in a semi-structured telephone interview, provided it is safe for them to do so, and they can be sure that the interview can take place without risk of being overheard. For male participants in the intervention arm, questions might include views on the recruitment process, motivation for joining the study, how those men who have started the intervention have found it so far and how they are dealing with the suspension of groups, how useful the “keeping-in-touch” calls from the programme coordinators are in this interim period, and how they are dealing with the impact of self-isolation. For men in the control arm, interviews will focus on the recruitment process, randomisation, and motivation to join the study as well as how they are dealing with the impact of self-isolation. Recruited (ex)partners will be asked if they feel their male (ex)partner’s behaviour has changed and how, whether they feel more or less safe and if the pandemic has had an impact on this, and whether they are sure they know where to get support if needed.
